# Small Noncoding RNAome Changes During Human Bone Marrow Mesenchymal Stem Cells Senescence *In Vitro*

**DOI:** 10.3389/fendo.2022.808223

**Published:** 2022-05-13

**Authors:** Fei Xiao, Jianping Peng, Yang Li, Xing Zhou, Ding Ma, Liming Dai, Jie Yuan, Xiaodong Chen, Chuandong Wang

**Affiliations:** ^1^ Department of Orthopedic Surgery, Xinhua Hospital Affiliated to Shanghai Jiao Tong University School of Medicine (SJTUSM), Shanghai, China; ^2^ Collaborative Innovation Centre of Regenerative Medicine and Medical BioResource Development and Application, Guangxi Medical University Nanning, Guangxi, China; ^3^ Department of Orthopaedic Surgery, The Second Hospital of Shanxi Medical University, TaiYuan, China

**Keywords:** bone marrow mesenchymal stem cells, senescence, miRNA, piRNA, snoRNA, snRNA

## Abstract

Bone marrow mesenchymal stem cells (BMSCs) have been used in stem cell-based therapy for various diseases due to their self-renewing ability and differentiation potential to various types of cells and immunoprivileged properties. However, the proliferation capability and functionality of BMSCs are known to decline with aging, which severely limits the extensive applications of BMSC-based therapies. To date, the exact mechanism involved in the cellular senescence of BMSCs remains unclear. RNA is thought to be the initial molecular form of life on earth. It also acts as a transmitter and important regulator of genetic information expression. There are many kinds of small noncoding RNAs with different functions in cells that regulate important life activity processes in multiple dimensions, including development process, gene expression, genomic stability, and cellular senescence. In this study, a replicative senescence model of hBMSCs was established and the expression changes of small noncoding RNAs during senescence were detected by small RNA high-throughput sequencing analysis and qPCR. Small RNA sequencing results showed that there were significant differences in the expression of 203 miRNAs, 46 piRNAs, 63 snoRNAs, 12 snRNAs, and 7 rasiRNAs. The results of qPCR, which was performed for the verification of the sequencing results, showed that there were significant differences in the expression of 24 miRNAs, 34 piRNAs, 34 snoRNAs, and 2 snRNAs. These findings might provide a novel insight into hBMSC senescence and contribute to the development of new targeting senescence strategies.

## Introduction

Bone marrow mesenchymal stem cells (BMSCs) are basic cells that are capable of self-renewal and multi-lineage differentiation into chondrocytes, osteoblasts, and adipocytes, which participate in the maintenance of the hematopoietic microenvironment (1). BMSCs are particularly important cells for the following reasons: first, they are the core members of bone formation and remodeling; second, they promote bone marrow tissue repair; in addition, they support the growth of hematopoietic progenitor cells in the immune and hematopoietic system. BMSCs are widely used in stem cell-based therapies and regenerative medicine. However, BMSCs comprise only a minimal fraction of marrow-nucleated cells, with an estimated frequency of only 0.001%–0.01% of nucleated cells (2). Approximately 10^8^ cells in total or 1×10^6^ cells/kg recipient weight are administered multiple times in most clinical trials using BMSCs, which requires a single stem cell to undergo at least 25 population doublings, so a substantial number of BMSCs with strong replication ability are needed (3). A previous study indicated that BMSCs were subjected to limitations in replicative life span and entered the replicative senescence stage between 50 and 90 days post-harvest (4). Earlier passage BMSCs have been demonstrated to have better clonogenic potential compared to later passage MSCs (5). Therefore, the development of *in vitro* expansion protocols is necessary to promote various clinical applications and essential manipulation of BMSCs. Consequently, it is important to understand the key molecular processes that control the proliferation and differentiation of BMSCs. Of all the factors related to the proliferation of BMSCs thus far identified, cellular senescence is undoubtedly an important one.

Recent studies have shown that small noncoding RNAs (SncRNAs), a class of important epigenetic regulatory molecules, play an important role in the regulating cellular senescence of mesenchymal stem cells (6–8). SncRNAs are short RNA species, typically fewer than 400 nucleotides in length, that are not translated into proteins but have other structural or functional biological roles (9). SncRNAs include microRNAs (miRNAs), small nuclear RNAs (snRNAs), small nucleolar RNAs (snoRNAs), P-element-induced wimpy testis (PIWI)-interacting RNAs (piRNAs), small interfering RNAs (siRNAs), transfer RNAs (tRNAs), and repeat-associated siRNAs (rasiRNAs). Some microRNAs have been reported to play a role in regulating the senescence of BMSCs. For example, miR-34a overexpression exacerbates while suppression alleviates rats’ BMSC replicative senescence and natural senescence by targeting Nampt (10). Overexpression miR-335 resulted in early senescence-like alterations in human BMSCs (hBMSCs) through inhibition of AP-1 activity (11). The expression and role of the other sncRNAs in the senescence of BMSCs remain unclear.

In the present study, hBMSCs were isolated and underwent ten additional passages to simulate replicative senescence *in vitro*. To gain insights into sncRNA changes in hBMSC senescence, we used small RNA sequencing (small RNA-seq) to generate extensive small RNA data and identified differentially expressed sncRNAs. These findings might provide novel insight into hBMSC senescence and contribute to the development of corresponding targeting anti-senescence strategies.

## Method and Materials

### Isolation and Culture of Bone Marrow Mesenchymal Stem Cells

The project has been approved by the ethics committee of Xinhua Hospital Affiliated with Shanghai Jiao Tong University School of Medicine (SJTUSM). The bone marrow was obtained from volunteer patients who accepted traumatic femoral neck fracture treatment by internal fixation surgery in the hospital. Informed consent was obtained from all donors. The upper fat drops were removed by centrifugation at 1500 r/min for 5 min, resuspend the remaining flushing solution and slowly inject it into the test tube pre-added with the same amount of lymphocyte separation solution (Percoll), centrifuge at 1500 r/min density gradient for 10 min, suck the middle monocyte layer, wash it twice with PBS, centrifuge at 1500 r/min for 5 min, discard the supernatant, precipitate, and use DMEM medium (containing 10% fetal bovine serum and 1% penicillin-streptomycin solution) to resuspend. The cells were inoculated into the culture plate, the culture medium was changed every 2 days, and the cells were subcultured after they grew into clonal clusters.

### β-Galactosidase Staining

According to the manufacturer’s instructions, the Cell Senescence β-Galactosidase Staining Kit [#40754ES60, Yesen Biotechnology (Shanghai)] was used for aging staining analysis of hBMSCs. The cells were washed twice with PBS, added with a fixing solution from the kit, and fixed at room temperature for 15 min. We removed the fixed solution and washed the cells three times with PBS for 3 min each time. We added 1 ml of preheated dyeing solution to each well to cover the whole growth surface. Incubation occurred at 37°C for 24 h. The positive cells were blue, as observed and counted under an ordinary light microscope.

### RNA Isolation and qPCR Detection

BMSCs were isolated from three patients for RNA extraction and qPCR detection. The patients were female, aged 19, 19, and 23 years, respectively. The liquid in the culture dish was discarded by the graduated straw, the PBS buffer was rinsed two times, 1 ml of Trizol reagent was added to the culture dish, and the cells were repeatedly beaten to make the cell lysis. The above cell lysate was transferred into an EP tube, 200 μl of chloroform was added, and the lysate was then placed on ice for 5–8 min. It was placed in a low-temperature high-speed centrifuge for centrifugation, the supernatant was transferred to a new EP tube, an equal volume of isopropanol was added to it, the RNA was precipitated, and it was placed on ice for 10 min. An equal volume of 75% ethanol was added, and it was immediately put into a low-temperature high-speed centrifuge for centrifugation. Pour out 75% ethanol. It was dried at room temperature, precipitated for 5–10 min, and the RNA was re-dissolved. The RNA was reverse transcribed into cDNA as described in the manufacturer’s instructions of PrimeScript™ RT Master Mix (#RR036A, TAKARA) and miRNA First Strand cDNA Synthesis [#B532451, Sangon Biotech (Shanghai)]. According to the manufacturer’s instructions of TB Green™ Premix Ex Taq™ II (Tli RNaseH Plus) (#RR042A, TAKARA), the liquid preparation of each component was carried out on ice and was operated away from light. After the liquid of each component was added into the EP tube, it was placed on the vortex oscillator for oscillation, and then the qPCR reactions were carried out. RNA from BMSCs of three patients were detected by qPCR, respectively. The standard PCR amplification procedure was as follows: Step 1: 95°C for 30 s; Step 2: 95°C for 5 s, 60°C for 30 s, 50 cycles; and Step 3: dissolution curve. After the cycle, the amplification and dissolution curves were analyzed, the expression values of different genes were recorded for each group, and then the expression of the target genes was recorded for each group according to the 2^−△△CT^ method. All primers used are listed in **Supplementary Materials S1**.

### Small RNA Sequence

The BMSCs isolated from a 23-year-old female patient were used for small RNA isolation, purification, cDNA library preparation, sequencing, and bioinformatics analysis, which were completed by SANGON biotech (Shanghai). The experimental operation was carried out in strict accordance with the reagent and instrument instructions. The main experimental processes include isolation of small RNA from total RNA, 5 ‘connector connection, 3’ connector connection, RT-PCR amplification, purification of small RNA library, detection of the library, cluster amplification of DNA in cluster station, sequencing on an Illumina Hiseq 2000 sequencer, and bioinformatics analysis of clean sequences with GenomeStudio software. Expression profiles of sncRNAs in two groups of cells were analyzed using the same samples.

### Statistical Analysis

All data were shown with mean ± S.D. of three independent experiments. *t*-test and ANOVA were used for comparing two groups by Excel 2017, and *p* < 0.05 was considered as a significant difference.

## Results

### Subculture Leads to hBMSC Senescence

**Figure 1:**
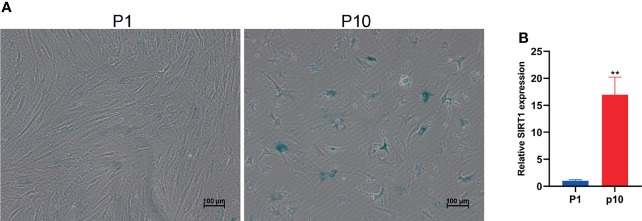
Subculture leads to hBMSC senescence. **(A)** The staining of β-galactosidase of hBMSCs at passage 1 (P1) and passage 10 (P10). **(B)** The relative expression of SIRT1 mRNA in hBMSCs at P1 and P10. ** indicated *p* < 0.01.

Cellular senescence is of great significance to individual health. Replicative senescence is an important form of senescence. In order to detect the expression changes of small RNA in the aging process of hBMSCs, we firstly established a replicative senescence model of hBMSCs. We subcultured hBMSCs and collected P1 and P10 hBMSCs, respectively. The microscopic observation results showed that compared with P1 group hBMSCs, the morphology of P10 hBMSCs shrank significantly (**Figure 1A**). The β-galactosidase activity assay showed that the β-galactosidase activity of hBMSCs in the P10 group increased significantly. The qPCR results showed that the expression of SIRT1 mRNA in hBMSCs in the P10 group was significantly higher than that in the P1 group (**Figure 1B**). Taken together, these results indicate that the replicate passaging of PBMCs can lead to cellular senescence and can be used for modeling marrow mesenchymal stem cell aging *in vitro*.

### Abnormal miRNA Expression During hBMSC Senescence

**Figure 2:**
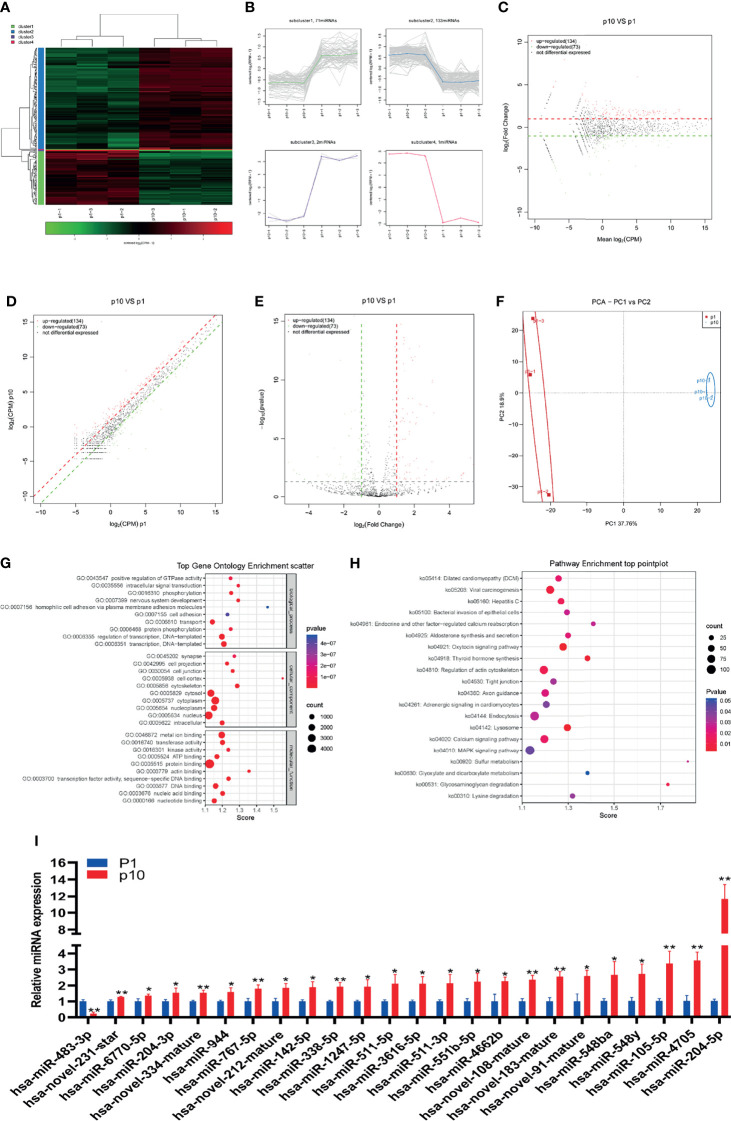
Abnormal miRNA expression during hBMSC senescence. **(A)** A heatmap showing the differential expression of miRNA in hBMSCs at P1 and P10. **(B)** A subcluster map showing the differential expression of miRNA in hBMSCs at P1 and P10. **(C)** An MA map showing the differential expression of miRNA in hBMSCs at P1 and P10. **(D)** A scatter map showing the differential expression of miRNA in hBMSCs at P1 and P10. **(E)** A volcano map showing the differential expression of miRNA in hBMSCs at P1 and P10. **(F)** A PCA map showing the differential expression of miRNA in hBMSCs at P1 and P10. **(G)** Gene ontology analysis in BMSCs at P1 and P10. **(H)** KEGG analysis in BMSCs at P1 and P10. **(I)** Differential expression of miRNA in hBMSCs at P1 and P10 detected by qPCR. * indicated *p* < 0.05, ** indicated *p* < 0.01.

The small RNA sequence showed differential expression of miRNA in P1 and P10 hBMSCs (**Figure 2A**). The differential expression of miRNA was divided into four subclusters (**Figure 2B**). There were 134 miRNAs upregulated and 73 miRNAs downregulated in hBMSCs from the P10 group compared to the P1 group hBMSCs, and the results are represented by MA, scatter, and volcano maps (**Figures 2C–E**). Principal component analysis (PCA) showed that the hBMSCs of P1 and P10 groups could be clearly distinguished based on the differential expression of miRNA (**Figure 2F**). Gene ontology analysis showed the GO enrichment in BMSC senescence (**Figure 2G** and **Supplementary Materials S2**). KEGG analysis showed the pathway enrichment in BMSC senescence (**Figure 2H** and **Supplementary Materials S3**). QPCR showed that 23 miRNAs were upregulated in hBMSCs of the P10 group compared with hBMSCs of the P1 group (**Figure 2I**). On the other hand, qPCR showed that only has-miR-483-3p were downregulated in hBMSCs of the P10 group compared with hBMSCs of the P1 group (**Figure 2I**). At the same time, we predicted the target genes of significantly differentially expressed miRNA (**Supplementary Materials S4**).

### Abnormal piRNA Expression During hBMSC Senescence

**Figure 3:**
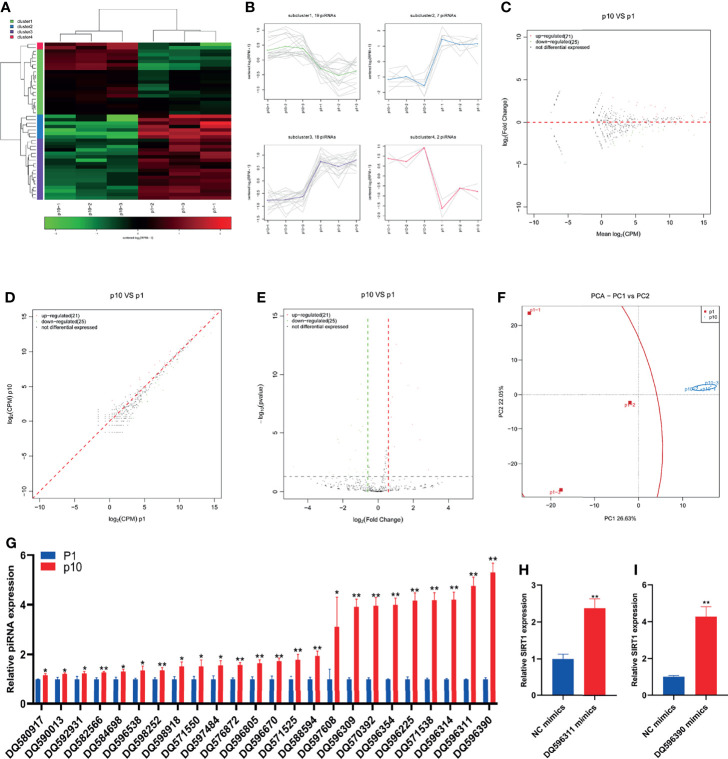
Abnormal piRNA expression during hBMSC senescence. **(A)** A heatmap showing the differential expression of piRNA in hBMSCs at P1 and P10. **(B)** A subcluster map showing the differential expression of piRNA in hBMSCs at P1 and P10. **(C)** An MA map showing the differential expression of piRNA in hBMSCs at P1 and P10. **(D)** A scatter map showing the differential expression of piRNA in hBMSCs at P1 and P10. **(E)** A volcano map showing the differential expression of piRNA in hBMSCs at P1 and P10. **(F)** A PCA map showing the differential expression of piRNA in hBMSCs at P1 and P10. **(G)** Differential expression of piRNA in hBMSCs at P1 and P10 detected by qPCR. **(H)** The relative expression of SIRT1 mRNA in hBMSCs with piRNA DQ596311 overexpression. **(I)** The relative expression of SIRT1 mRNA in hBMSCs with piRNA DQ596390 overexpression. * indicated *p* < 0.05, ** indicated *p* < 0.01.

Even though many years have passed since the discovery of piRNA in many species, we still do not know how to start the response of new piRNA and the accurate function of piRNA during development, and there are still many open problems to be solved urgently. Due to a large number of piRNAs, the identification of piRNAs is more challenging than miRNAs. However, with the improvement of chip and high-throughput sequencing technology, researchers will be able to conduct a comprehensive and in-depth study on the biological function of piRNA. The small RNA sequence showed differential expression of piRNA in P1 and P10 hBMSCs (**Figure 3A**). The differential expression of piRNA was divided into four subclusters (**Figure 3B**). There were 21 upregulated piRNAs and 25 downregulated piRNAs in hBMSCs of the P10 group compared to the P1 group hBMSCs, and these are represented by MA, scatter, and volcano maps (**Figures 3C–E**). PCA showed that the differential expression of piRNA could clearly distinguish between the hBMSCs of the P1 and P10 groups (**Figure 3F**). QPCR showed that 24 piRNAs were upregulated in hBMSCs of the P10 group compared with hBMSCs of the P1 group (**Figure 3G**). On the other hand, we had not found piRNAs that were downregulated in hBMSCs of the P10 group compared with hBMSCs of the P1 group detected by qPCR (**Figure 3G**). PiRNA DQ596311 and DQ596390 were overexpressed in P1 hBMSCs using piRNA DQ596311 and DQ596390 mimics. We found that overexpression of piRNA DQ596311 and DQ596390 could promote the mRNA expression of aging-related gene SIRT1 (**Figures 3H, I**).

### Abnormal snoRNA Expression During hBMSC Senescence

**Figure 4:**
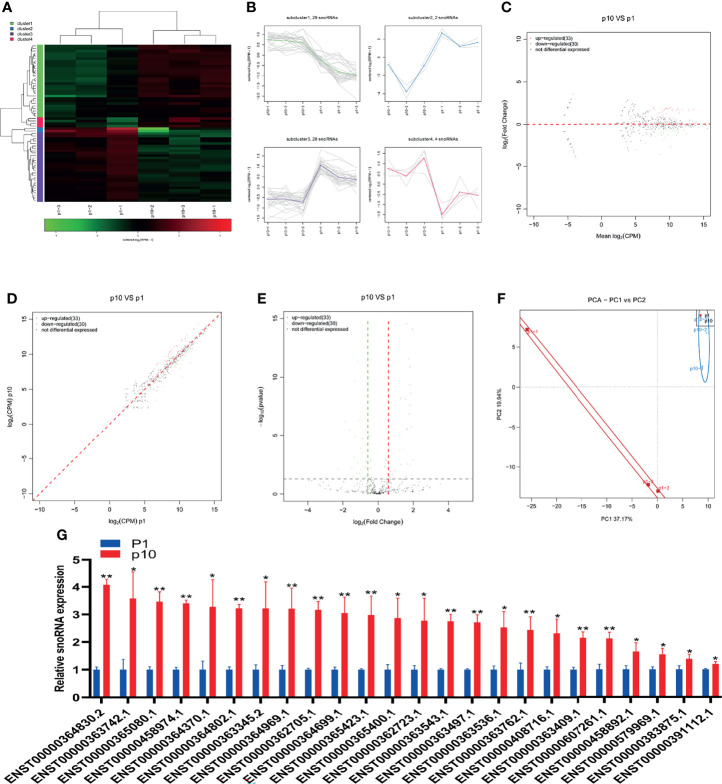
Abnormal snoRNA expression during hBMSC senescence. **(A)** A heatmap showing the differential expression of snoRNA in hBMSCs at P1 and P10. **(B)** A subcluster map showing the differential expression of snoRNA in hBMSCs at P1 and P10. **(C)** An MA map showing the differential expression of snoRNA in hBMSCs at P1 and P10. **(D)** A scatter map showing the differential expression of snoRNA in hBMSCs at P1 and P10. **(E)** A volcano map showing the differential expression of snoRNA in hBMSCs at P1 and P10. **(F)** A PCA map showing the differential expression of snoRNA in hBMSCs at P1 and P10. **(G)** Differential expression of snoRNA in hBMSCs at P1 and P10 detected by qPCR. * indicated *p* < 0.05, ** indicated *p* < 0.01.

SnoRNA is a kind of small non-coding RNA showing a high abundance in the nucleus. In the last 10 years, a large number of new snoRNAs have been identified from a variety of model organisms by RNAomics. The results show that snoRNA participates in the processing and folding of rRNA precursors and has the function of guiding rRNA, tRNA, and snRNA post-transcriptional nucleoside modification. The small RNA sequence showed differential expression of snoRNA in P1 and P10 hBMSCs (**Figure 4A**). The differential expression of snoRNA was divided into four subclusters (**Figure 4B**). There were 33 upregulated snoRNAs and 30 downregulated snoRNAs in hBMSCs of the P10 group compared to the P1 group hBMSCs, and these are represented by MA, scatter, and volcano maps (**Figures 4C–E**). PCA showed that the differential expression of snoRNA could clearly distinguish between the hBMSCs of the P1 and P10 groups (**Figure 4F**). QPCR showed that 24 snoRNAs were upregulated in hBMSCs of the P10 group compared with hBMSCs of the P1 group (**Figure 4G**). On the other hand, we had not found snoRNAs that were downregulated in hBMSCs of the P10 group compared with hBMSCs of the P1 group detected by qPCR (**Figure 4G**).

### Abnormal snRNA Expression During hBMSC Senescence

**Figure 5:**
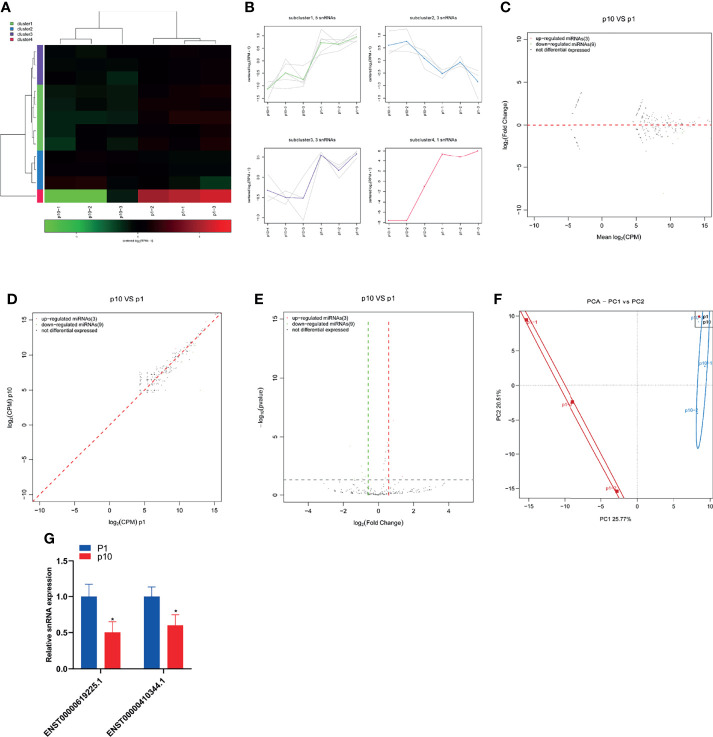
Abnormal snRNA expression during hBMSC senescence. **(A)** A heatmap showing the differential expression of snRNA in hBMSCs at P1 and P10. **(B)** A subcluster map showing the differential expression of snRNA in hBMSCs at P1 and P10. **(C)** An MA map showing the differential expression of snRNA in hBMSCs at P1 and P10. **(D)** A scatter map showing the differential expression of snRNA in hBMSCs at P1 and P10. **(E)** A volcano map showing the differential expression of snRNA in hBMSCs at P1 and P10. **(F)** A PCA map showing the differential expression of snRNA in hBMSCs at P1 and P10. **(G)** Differential expression of snRNA in hBMSCs at P1 and P10 detected by qPCR. * indicated *p* < 0.05.

SnRNA is the main component of RNA spliceosome in the post-transcriptional processing of eukaryotes. Its length is about 100–215 nucleotides in mammals. SnRNA has always existed in the nucleus. It forms RNA spliceosomes with about 40 nuclear proteins and plays an important role in RNA post-transcriptional processing. The small RNA sequence showed differential expression of snRNA in P1 and P10 hBMSCs (**Figure 5A**). The differential expression of snRNA was divided into four subclusters (**Figure 5B**). There were three upregulated snoRNAs and nine downregulated snoRNAs in hBMSCs of the P10 group compared to P1 group hBMSCs, and these are represented by MA, scatter, and volcano maps (**Figures 5C–E**). PCA showed that the differential expression of snRNA could clearly distinguish between the hBMSCs of the P1 and P10 groups (**Figure 5F**). qPCR results showed that two snRNAs were downregulated in hBMSCs of the P10 group compared with hBMSCs of the P1 group (**Figure 5G**). On the other hand, we did not find snRNAs that were upregulated in the hBMSCs of the P10 group compared with hBMSCs of the P1 group detected by qPCR (**Figure 5G**).

### Abnormal rasiRNA Expression During hBMSC Senescence

**Figure 6:**
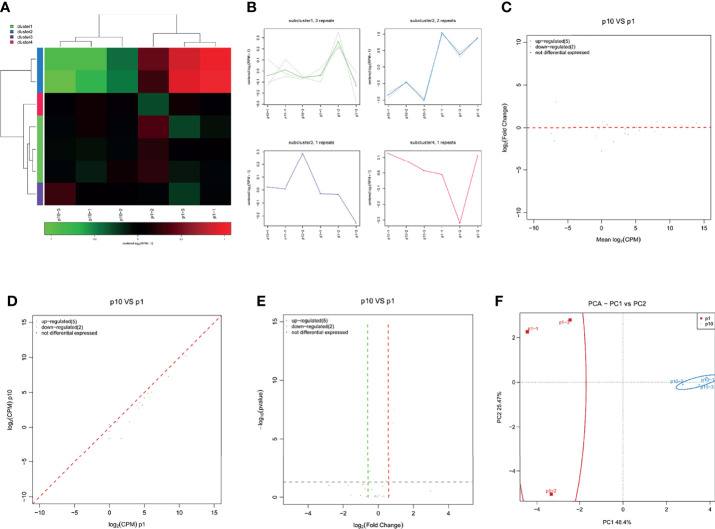
Abnormal rasiRNA expression during hBMSC senescence. **(A)** A heatmap showing the differential expression of rasiRNA in hBMSCs at P1 and P10. **(B)** A subcluster map showing the differential expression of rasiRNA in hBMSCs at P1 and P10. **(C)** An MA map showing the differential expression of rasiRNA in hBMSCs at P1 and P10. **(D)** A scatter map showing the differential expression of rasiRNA in hBMSCs at P1 and P10. **(E)** A volcano map showing the differential expression of rasiRNA in hBMSCs at P1 and P10. **(F)** A PCA map showing the differential expression of rasiRNA in hBMSCs at P1 and P10.

RasiRNA was first found in the embryonic development of *Drosophila melanogaster* and zebrafish and recently found in *Drosophila* germline cells. The expression of rasiRNA is highly enriched in the testes and early embryos. The length of rasiRNA is 22–24 nt, which is similar to transposable factor, satellite, and microsatellite DNA and the stellate repeat inhibitor gene. RasiRNA also binds to piwi proteins, which may silence retrotransposons and repeat elements in germ cells to regulate the development of *Drosophila* germline. Its silencing mechanism may be related to the regulation mode of piRNA. The small RNA sequence showed differential expression of rasiRNA in P1 and P10 hBMSCs (**Figure 6A**). The differential expression of rasiRNA was divided into four subclusters (**Figure 6B**). There were five upregulated rasiRNAs and two downregulated rasiRNAs in the hBMSCs of the P10 group compared to P1 group hBMSCs, and this is represented by MA, scatter, and volcano maps (**Figures 6C–E**). PCA showed that the differential expression of rasiRNA could clearly distinguish between the hBMSCs of the P1 and P10 groups (**Figure 6F**).

## Discussion

Although BMSCs have strong self-renewal ability, as the number of passages increases, hBMSCs cultured *in vitro* gradually present the characteristics of senescent cells such as permanent proliferative arrest and the release of harmful pro-inflammatory molecules and growth factors as part of the senescence-associated secretory phenotype (SASP) (12, 13). In addition, the accumulation of senescent cells disordered tissue function and metabolism (14). These unfavorable factors due to cellular senescence obviously limit the application of stem cell-based therapy. Thus, it is of great significance to explore the mechanism of cellular senescence and find the potential anti-senescence targets.

SncRNAs, a large class of regulatory molecules, are involved in organism development and coordination of biological processes, including metabolism, maintaining genome integrity, and immune and stress responses (15). Recent studies have suggested that sncRNAs play an important role in regulating the senescence of mesenchymal stem cells (9, 12, 16, 17). The aim of this study was to systematically investigate the sncRNA profile changes in senescent hBMSCs after long-term expansion *in vitro*. We found that hBMSCs at passage 10 showed obvious senescence characteristics, including increased SA-β-Gal activity and senescence-related gene expression. Moreover, the expression profiles of miRNAs, snRNAs, snoRNAs, piRNAs, and rasiRNAs in passage 10 hBMSCs were significantly different from those in passage 1.

MiRNAs, the most studied sncRNAs, exert their negative regulatory functions at the post-transcriptional level by partially or fully binding to the untranslated region at the 3’ end of target gene mRNA, representing a new level of gene expression regulation. The sequencing and qPCR results show that there were 23 miRNAs upregulated while one miRNA was downregulated in passage 10 hBMSCs. Some miRNAs that we determined were altered with subculture passage in hBMSCs have been demonstrated in previous studies to play a role in suppressing osteogenesis and inducing cellular senescence. For example, in the chondrocytes of aging mice induced by ionizing radiation or DNA damaging chemical agents, the expression of miR-204 is significantly upregulated, and has important regulatory effects on SASP factors such as IL-6 and MMP-3 (18). In addition, miR-204 can inhibit the osteogenic differentiation of MSCs by targeting Runx2 (19). In the present study, miR-204 shows the most upregulated extent in passage 10 hBMSCs; the finding implies that miR-204 may play an important role in the senescence process of hBMSCs. It is reported that miR-183 increased during cellular senescence after exposure to oxidative stress (20), and suppressed the osteogenic differentiation in BMSCs (21). Our result shows that miR-183 increases with senescence, consistent with these previous findings. Since the function of the other differentially expressed miRNAs in senescent hBMSCs remains unclear, further studies are needed.

PiRNAs are another type of small RNAs with approximately 24–31 nucleotides in length. The precursors of piRNAs are derived from tandem repeat sequences called piRNA clusters and form a mature piRNA/PIWI complex *via* two route-dependent or independent “Ping-Pong” amplification pathways (22). In the past, it was believed that piRNAs existed only in the reproductive system to regulate the growth and development of germ cells. A recent study indicated that piRNAs are also expressed in several human tissues with tissue specificity (23). PiRNAs play roles in transcriptional or posttranscriptional gene silencing pathways by combining Piwi subfamily proteins. PiRNA research mainly focuses on its regulation of the occurrence and development of cancer, including colorectal cancer, breast cancer, and lung cancer (23). The latest research shows that piRNA-36741 was upregulated during the osteogenic differentiation of hBMSCs. Administration with piR-36741 mimic attenuated ovariectomy-induced osteoporosis in mice through METTL3-mediated m6A methylation of BMP2 transcripts (24). To our knowledge, there are no reports on the relationship between piRNAs and hBMSC senescence. Our results show that 24 piRNAs are upregulated in hBMSCs of the P10 group compared with hBMSCs of the P1 group and no downregulated piRNA was verified by qPCR. Further studies are needed to clarify the function of differentially expressed piRNAs.

SnoRNAs, a class of evolutionarily conserved noncoding small guide RNAs with lengths of 60–300 nucleotides, are extensively studied noncoding RNAs that primarily accumulate in the nucleoli (25). SnoRNAs are involved in direct chemical modification of other RNA substrates and the regulation of alternative splicing and post-transcriptional modification of snRNA, tRNA, and mRNA, while others exhibit microRNA-like activity (26). SnRNA is the main component of the RNA spliceosome in the posttranscriptional processing of eukaryotes and participates in splicing pre-mRNA and noncoding transcripts in cells. In humans, its length is approximately 60–330 nucleotides, and it can be divided into 14 categories. Rn7SK is a highly conserved snRNA transcribed by RNA polymerase III and is a regulator of Pol II activity through the inhibition of the function of positive transcriptional elongation factor b. A recent study demonstrated that Rn7SK participates in the regulation of cellular senescence, with the transient knockdown of Rn7SK in MSCs leading to delayed senescence, while its overexpression shows the opposite effects (7). However, the role of snoRNAs and snRNAs in hBMSCs and their relationship with senescence has not yet been reported. The functions and potential targets of these snRNAs in chondrocytes will be explored and verified in our subsequent studies. However, many sequencing results for snoRNAs and snRNAs are inconsistent with the expression trends observed *via* qRT-PCR. We speculate that there may be many stem-loop structures in snoRNAs and snRNAs that will affect the sequencing or qRT-PCR results. The sequencing results showed that rasiRNAs were differentially expressed in senescent hBMSCs. However, due to the scarcity of research on rasiRNAs and its short length, it is difficult to verify the sequencing results by conventional real-time PCR. More studies are needed to explore the role of rasiRNAs in hBMSC senescence.

## Conclusion

In this study, we detected the expression changes in miRNAs, piRNAs, snoRNAs, snRNAs, and rasiRNAs during hBMSC senescence by high-throughput small RNA sequencing and detected the expression of miRNAs, piRNAs, snoRNAs, and snRNAs during hBMSC senescence by qRT-PCR. We found that the expression of miRNA, piRNA, snoRNA, snRNA, and rasiRNA changes significantly during hBMSC senescence.

## Data Availability Statement

The datasets presented in this study can be found in online repositories. The names of the repository and accession number can be found below: The data has been uploaded to GEO database and the accession number is GSE192578.

## Ethics Statement

The studies involving human participants were reviewed and approved by the Ethics committee of Xinhua Hospital Affiliated with Shanghai Jiao Tong University School of Medicine (SJTUSM). The patients/participants provided their written informed consent to participate in this study.

## Author Contributions

Conceptualization: XC and CW. Methodology: FX, JP, and YL. Software: XZ, DM, LD, and JY. Data curation: JP and YL. Writing, review, and editing: FX and CW. All authors contributed to the article and approved the submitted version.

## Funding

This work is supported by Shanghai Sailing Program (20YF1429100 and 19YF1431200), National Natural Science Foundation of China (82172473 and 81802191), Natural Science Foundation of Shanghai (19ZR1433100), Interdisciplinary of Medicine and Engineering Foundation of Shanghai Jiao Tong University (YG2019ZDA22), and Biomedical Technology Support Program of Shanghai (20S31900200). Natural Science Foundation of Shandong Province (ZR2019PH068).

## Conflict of Interest

The authors declare that the research was conducted in the absence of any commercial or financial relationships that could be construed as a potential conflict of interest.

## Publisher’s Note

All claims expressed in this article are solely those of the authors and do not necessarily represent those of their affiliated organizations, or those of the publisher, the editors and the reviewers. Any product that may be evaluated in this article, or claim that may be made by its manufacturer, is not guaranteed or endorsed by the publisher.
